# Family planning behaviours among women with diabetes mellitus: a scoping review

**DOI:** 10.1186/s40001-023-01626-1

**Published:** 2024-01-11

**Authors:** Sarah Awang Dahlan, Idayu Badilla Idris, Azmawati Mohammed Nawi, Rahana Abd Rahman

**Affiliations:** 1 Department of Public Health Medicine, Faculty of Medicine, Universiti Kebangsaan Malaysia, 56000 Kuala Lumpur, Malaysia; 2 Family Health Development Division, Ministry of Health, Complex E, 62590 Putrajaya, Malaysia; 3 Department of Obstetrics and Gynaecology, Faculty of Medicine, Universiti Kebangsaan Malaysia, 56000 Kuala Lumpur, Malaysia

**Keywords:** Family planning, Contraception, Diabetes mellitus, Preconception care

## Abstract

**Introduction:**

Diabetes mellitus in pregnancies is associated with adverse outcomes both for the mothers and babies. Postponing pregnancy in unoptimized conditions and stabilisation of glucose should be prioritized. This scoping review is aimed to determine the scope and at the same time map the types of evidence available that is related to family planning behaviours among women with diabetes mellitus, with a particular focus on their factors which influence family planning usage and subsequently enable the identification of knowledge gaps in preventing unintended pregnancies among this high-risk population.

**Methods:**

This scoping review is guided by the methodological framework by Arksey and O’Malley’s and Prisma-ScR checklist. PubMed, EBSCO and OVID were searched for empirical studies between 2000 and February 2022 using the search terms “family planning”, “contraceptive” and “diabetes mellitus”. Data were summarized according to the study characteristics and levels of factors influencing family planning behaviours.

**Results:**

Thirty-five articles that met the eligibility criteria included 33 quantitative studies, one qualitative study and one mixed-methods study. The prevalence of family planning methods used by women with diabetes mellitus varied ranging from 4.8 to 89.8% among the studied population. Women with diabetes mellitus were reported to be less likely to utilise any family planning methods compared to women without diabetes mellitus.

**Conclusions:**

Most of the evidence to date on family planning behaviours among women with diabetes mellitus focuses on the role of individual level sociodemographic factors. Few studies focused on exploring determinants at multiple levels. In this review we found that there is limited evidence on disease control and pregnancy intention in relation to their family planning practices. Future studies with more clinical and contextual factors are needed to guide the strengthening of family planning services for high-risk group women specifically for women with diabetes mellitus.

**Supplementary Information:**

The online version contains supplementary material available at 10.1186/s40001-023-01626-1.

## Abstract (plain language summary)

As the prevalence of diabetes mellitus is increasing globally, more women in reproductive age group are living with diabetes mellitus. Pregnant women with uncontrolled diabetes mellitus have higher risk for complications, both to the mothers and the baby. Therefore, it is very important that family planning needs of women with diabetes mellitus are met. This review is aimed to identify what is known and not known about the factors influencing family planning behaviours among women with diabetes mellitus. We searched three databases for studies published from 2000 to February 2022. Our review included 35 articles and nearly all of the studies were quantitative, with one qualitative and one mixed-methods study. Among the studies that compared between women with diabetes mellitus and without diabetes mellitus, less women with diabetes mellitus were using family planning. Some papers also include the reasons or barriers for using family planning among the studied population. Majority of the articles described sociodemographic were factors related to family planning usage, while only few studies explored beyond individual factors. Little information on the clinical profile of the women with diabetes mellitus were known. Future research should examine clinical and other non-individual factors influencing family planning among this particular group of women as sexual and reproductive health in general is very much influenced by cultural or healthcare system factors.

**Supplementary Information:**

The online version contains supplementary material available at 10.1186/s40001-023-01626-1.

## Background

Diabetes mellitus is the most common medical conditions complicating pregnancy which has been reported in several countries including in the United Kingdom, United States of America and Australia [1–3]. Reports from these countries are showing increasing prevalence of pregnancies affected by diabetes throughout the years, in line with the increasing trend of diabetes prevalence among the general population globally [4, 5]. Diabetes in pregnancy is either pre-existing diabetes, where the diagnosis is prior to conception, or gestational diabetes, where diabetes is first detected during pregnancy. It has been established that pregnancies with diabetes mellitus are associated with adverse outcomes both to the mothers and babies compared to pregnancies without diabetes mellitus, including increased risk of pre-eclampsia, congenital malformations, spontaneous miscarriage, premature birth, foetal growth restriction, stillbirth, neonatal hypoglycaemia and birth trauma due to foetal macrosomia [6]. Pregnant women with pre-existing diabetes-related micro-vasculopathy are also at higher risk of disease progression [7]. Comparable perinatal outcome has been seen between pregnancies with and without diabetes mellitus when the mothers achieved satisfactory disease control [8]. Therefore, postponing pregnancy in suboptimized conditions and stabilisation of diabetic control among women with pregnancy intention should be prioritized. Contraception advice is one of the preconception care components that should be provided to all women with diabetes mellitus within the reproductive age group [9–12] especially when a significant proportion of pregnancies around the world were unintended [13]. The higher percentage of unintended pregnancies among women with diabetes is also worrying as it represented a missed opportunity to provide preconception care which includes provision of counselling regarding the risks associated with diabetes mellitus during pregnancy, medication review to avoid possible teratogenic agents, folic acid supplementation, diet and weight loss advice, as well as screening for other diabetes-related complications [14, 15]. Extensive literatures are available on determinants of family planning behaviours among women of general population but in-depth evidence for women with specific condition such as diabetes mellitus are still minimal. There is no previous systematic review on family planning among women with diabetes mellitus. Women with risk factors such as diabetes mellitus may have different aims for family planning compared to women in the general population which are mainly to provide spacing and limit the number of children. This warrants for a review of all the evidence available related to family planning behaviours among women with diabetes mellitus to identify knowledge gaps with the ultimate aim of preventing unintended pregnancies among women with diabetes mellitus which is associated with higher risk of morbidity and mortality.

This scoping review is mainly aimed to determine the scope and map the types of evidence available related to family planning behaviours among women with diabetes mellitus and its determinants. This review also hopes to identify and analyse knowledge gaps in the family planning behaviours of women with diabetes mellitus in preventing unintended pregnancies which is associated with higher risk of morbidity and mortality.

## Materials and methods

This scoping review was guided by methodological framework by Arksey and O’Malleys [16] guided by Prisma-ScR checklist as outlined by Tricco [17] which includes identifying the research question, identifying the relevant studies, study selection, charting the data, and collating, summarising and reporting the results.

Scoping review methodology was selected to reflect our broad objectives to map the available evidence related to family planning behaviours among women with diabetes mellitus and eventually to identify and analyse the gaps in knowledge surrounding this topic. To identify the research question, Population-Concept-Context (PCC) framework (Table 1) as recommended by Joanna Briggs Institute for Scoping Reviews was used to outline the key elements of the review [18] which led to our main question ‘What is the available evidence surrounding family planning usage or practice among women with diabetes mellitus’. After the initial literature search, a specific sub-question was identified, which is to determine the factors influencing the usage.

**Table 1:** PCC Framework

Population	Reproductive-aged women with diabetes mellitus
Concept	Family planning methods or contraceptive usage
Context	Global studies

### Protocol and registration

Our protocol is drafted based on Preferred Reporting Items for Systematic reviews and Meta-Analyses extension for Scoping Reviews (PRISMA-ScR) [17], which was revised by the research team. The final protocol is registered with the Open Science Framework (https://osf.io/kv9hu/).

#### Eligibility criteria

To answer our research questions and achieve the objectives of the review, studies mentioning family planning or contraceptive usage among women with diabetes mellitus were included in the review. All studies including quantitative, qualitative and mixed-methods studies published in peer-reviewed journal were included to consider contextual factors influencing family planning behaviours. Studies from 2000 until February 2022 were selected as the wider duration hoped to allow any changes in trends of emerging evidences surrounding this topic. We excluded reviews and limited our studies to empirical research to focus on new knowledge and prevent redundancy. Studies were excluded if they do not contain evidence of family planning behaviours among women with diabetes mellitus and if they were not available in English (Table 2).

**Table 2:** Selection criteria for studies to be included in the review

Inclusion criteria	Exclusion criteria
• Available in English • Published between year 2000 until February 2022 • Empirical studies including quantitative, qualitative and mixed-methods studies • Studies conducted from low-middle income countries (LMICs) and high-income countries • Studies reporting family planning behaviours among women with diabetes mellitus within reproductive age group (15–49 years old) as the outcome or dependent variable	• Review paper • Clinical trials or studies on clinical efficacy or safety of contraceptives

### Information sources and search strategy

The first phase of the review involved identifying keywords and filters to be used in database search. Articles were identified using the following search terms: “family planning”, “contraceptive” and “diabetes mellitus”. Boolean terms such as “AND” and “OR” were used to separate keywords.(Additional File 1 for search strategy) Articles or relevant documents were identified by searching databases including PubMed, EBSCOhost, OVID and Web of Science Core Collection. Two different platforms searching MEDLINE database (i.e.; EBSCOhost and OVID) were used to ensure the breadth of the search as there might be variations in indexing that influence the search yield. Search was carried out according to PRISMA guidelines. The keywords searched is included in Table 3 below. Search was limited for specific field (title and/or abstracts) to capture only highly relevant articles. Articles were also searched by analyzing the reference lists of all the identified studies for additional articles or grey literature.

**Table 3:** Keyword searches

Date searched	Keyword search terms	Database	Number of studies
13 March 2022	Contraception OR birth control OR family planning OR contraceptive AND diabetes	MEDLINE Complete (EBSCOhost)	100
16 March 2022	“Family planning” [Title/Abstract] OR “Contraception” [Title/Abstract] OR “Contraceptive” [Title/Abstract]) AND “Diabetes” [Title/Abstract]	PubMed	762
18 March 2022	(Contracepti* or 'family planning') and diabetes	MEDLINE (OVID)	585
25 August 2023	“Family planning” OR “contraceptive” OR “Contraception” and “diabetes” OR “Diabetic”	Web of Science Core Collection	796

### Study selection process

The search results were imported into reference manager (Mendeley) where they were screened for duplicates. Three reviewers independently evaluated the titles, abstracts and then full text of all articles identified based on the eligibility criteria described above to minimise reporting bias as recommended by Joanna Briggs’s Institute [19]. Disagreements or inconsistencies among reviewers were resolved by discussion with other reviewers. Since the aim of the review is to study the family planning behaviour of women with diabetes mellitus, we excluded clinical trials or papers that focused on clinical efficacy or safety of contraceptive methods.

### Data charting process

Information from the selected studies were extracted into data-charting form in Microsoft Excel which contains the following variables; authors, year of publication, title of study, country of study, objectives of study, study methods and design, study settings, sample size, types of study participants and outcomes or factors studied in the study. Data were charted independently by each reviewer simultaneously followed by discussions to determine consistency of data extraction to answer our objectives and research questions. Data-charting forms were updated continuously in an iterative process.

### Data items

Data extracted includes articles’ characteristics (e.g., country of the study, study design, study population, study settings), types of information available on the main outcome (e.g., types of family planning methods, effectiveness of methods used, methods’ duration of action) and levels or categories of determinants measured (e.g., sociodemographic factors, clinical factors, interpersonal factors, institutional factors, community factors, policy factors). This categorization is based on Socioecological Model which was frequently adopted in previous systematic reviews describing family planning usage among general population [20–24]

### Synthesis of results

Once the data extraction was completed, content analysis of the extracted data was conducted. Studies were grouped based on study designs, types of study participants and outcomes measured. Quantitative and qualitative information were analyzed separately at first. Quantitative findings were collated based on association of explanatory variables or determinants in different studies. The explanatory variables were summarized according to its statistical significance whether positively or negatively associated with family planning behaviour. Qualitative findings were organized into main themes and further analyzed to identify contextual factors that influenced family planning behaviour among women with diabetes mellitus.

## Results

### Selection of sources of evidence

**Fig. 1:**
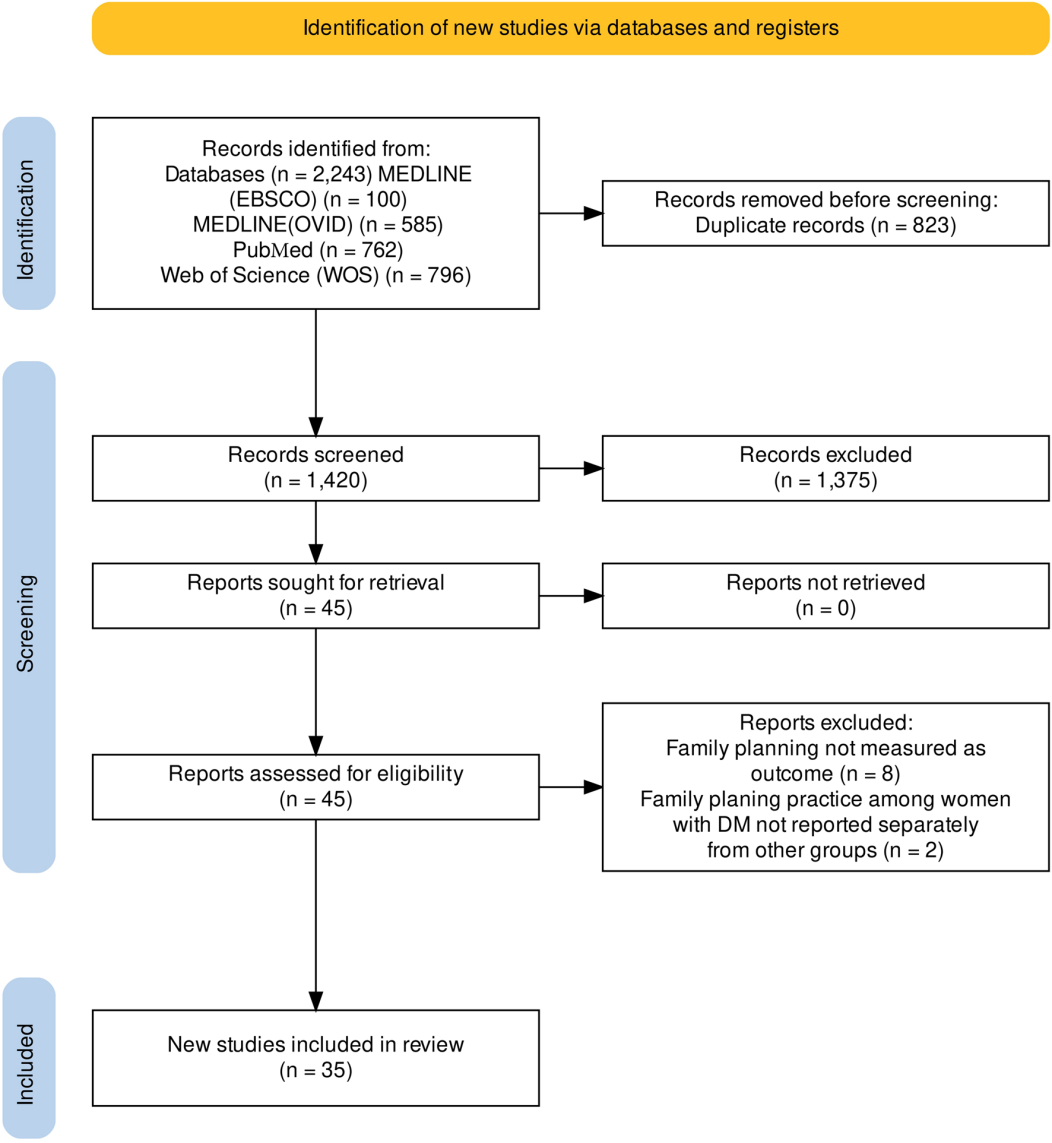
PRISMA flow diagram [31, 32]

A total of 1,447 articles were identified through our database search. After exclusion of duplicates, screening of abstracts and assessment of eligibility criteria, 35 articles that met the eligibility criteria were included in the review (Fig. 1). Most articles were excluded during the initial screening because they were reviews on contraceptive recommendations for women with diabetes mellitus and studies were focused on the effect of hormonal contraceptives on diabetes mellitus and metabolic profiles. Eight articles were excluded after assessing the full texts due to following reasons:

1. Studies did not report family planning behaviours as the outcome [25–28]
2. Studies did not report family planning practice among women with diabetes mellitus separately from other groups [29, 30]

### Characteristics of sources evidence

The majority of the studies (*n* = 20) [25, 33–51] were conducted in the United States. The rest of the studies were conducted in Malaysia (*n* = 3) [52–54], United Kingdom (*n* = 4) [55–58], Iran (*n* = 2) [59, 60], Italy (*n* = 1) [61], Australia (*n* = 1) [62], France (*n* = 1) [63], South Africa (*n* = 1) [64], Ethiopia (*n* = 1) [65] and Jamaica (*n* = 1) [66]. Table 4 provides summary of the studies included in the review. Only one study was a qualitative study [52] and one study utilised mixed-methods approach [58]. The studies were spread out from 2003 until January 2022. Six studies utilized secondary data from pre-existing survey database [25, 37, 44, 46, 51] where three of them were nationwide survey [25, 44, 51], two were state-wide [45, 46] and one was combined from multiple states survey [37]. Five studies used insurance claim records [34, 38, 42, 47, 48], one study used a general practice database [57] and one study used a tertiary centre database [39]. Other studies were based on primary data collection. Sample size of the studies ranged from 12 to 7.5 million where the bigger sample sizes were usually from nationwide data that also included women in the general population in the analysis.

**Table 4:** Summary of characteristics of the studies

Author, year	Country	Title	Study objectives	Study design	Study setting	Study population and sample size (n)
Falsetti et al. [54]	United States	Condom Use, Pregnancy and STDs in Adolescent Females with and without Type 1 DM	To describe and compare adolescent females with and without diabetes in terms of use of condoms, pregnancy outcomes and sexually transmitted disease outcome	Case–control study	Multicentre – 4 major university-based diabetic clinics	Adolescent females aged 16 to 22 years old with T1DM and without diabetes (*n* = 132)
Chuang et al. [37]	United States	Contraceptive Use by Diabetic and obese women	To describe contraceptive use by diabetic and overweight/obese women compared to women without these conditions	Cross-sectional—surveillance system	11 states (Family Planning Module of the Behavioral Risk Factor Surveillance System)	Sexually active women aged 18–44 who were not trying to conceive (*n* = 7,943)
Diabetes and Pregnancy Group, [63]	France	Knowledge about preconception care in French women with type 1 diabetes	To assess the knowledge about preconception care among wmen with T1DM of childbearing age	Cross-sectional study	Multicentre – 11 Diabetes Centres	women of childbearing age with T1DM (*n* = 138)
Napoli et al. [61]	Italy	Contraception in diabetic women: an Italian study	To determine the pattern of contraception used by diabetic women in relation to their sociocultural background	Cross-sectional study	12 centres throughout the country	Fertile women with T1DM &T2DM (*n* = 667)
Charron-Prochownik et al. [35]	United States	Knowledge, attitudes and behaviors related to sexuality and family planning in adolescent women with and without diabetes	To examine knowledge, attitudes, intentions, and behaviours regarding diabetes and reproductive issues, sexual activity, and birth control in teens with diabetes in relation to a non-diabetic group	Case–control study	Multisite – 4 major universities	Adolescents aged 16–23 years old with diabetes and without diabetes (*n* = 117)
Shawe et al. [57]	United Kingdom	Use of hormonal contraceptive methods by women with diabetes	To establish use of hormonal contraception in UK women aged between 15 and 44 years with T1DM or T2DM compared with comparison groups with no DM	Cross-sectional study	General practice database across UK	Women with diabetes aged 15 to 44 years old (*n* = 1,312)
Mazaheri et al. [59]	Iran	Pattern of Contraceptive Use in Diabetic Women	To determine the patterns of contraceptive use among diabetic women referring to Ardabil Medical Sciences University in Iran	Cross-sectional study	Diabetes clinic of a university hospital (referral centre)	Women with diabetes aged 15–49 years (*n* = 100)
Vahratian et al. [51]	United States	Family-Planning Practices Among Women with Diabetes and Overweight and Obese Women in the 2002 National Survey for Family Growth	To examine contraceptive practices among diabetic women and obese women	Population survey	Nationwide survey National Survey for Family Growth	Women 20–44 years old (*n* = 5,955)
Schwarz et al. [49]	United States	Perceived access to contraception among adolescents with diabetes—barriers to preventing pregnancy complications	To assess beliefs, perceived access to, and practices regarding contraception among adolescent women with T1DM	Cross-sectional study	2 university-based diabetes centres	T1DM aged 13–19 years old from endocrinology practices (*n* = 89)
Schwarz et al. [48]	United States	Provision of Contraceptive Services to Women with Diabetes Mellitus	To compare rates of provision of contraceptive services among women with DM and women without chronic medical conditions	Retrospective cohort study	Integrated managed care organization in Northern California	15–44 years old women who had continuous membership and pharmacy benefits in a managed care organisation (*n* = 459,181) (8,182 women with diabetes)
Shawe et al. [58]	United Kingdom	Use of contraception by women with type 1 or type 2 diabetes mellitus: ‘ It’s funny that nobody really spoke to me about it’	1) To understand factors which promote or discourage use of contraception in women with DM 2) To identify contraceptive knowledge, skills and attitudes of health professionals involved in diabetes care	Mixed-method study	4 diabetes centres	Women aged 16 – 44 years with DM (*n* = 107 for quantitative, *n* = 16 for qualitative)
Manaf et al. [54]	Malaysia	Contraceptive Use among Women with Chronic Medical Conditions and Factors Associated with Its Non-Use in Malaysia	To describe contraceptive use among women with chronic medical conditions and factors associated with its non-use	Cross-sectional study	3 medical specialist outpatient clinic in hospitals and 8 health clinics	Women with chronic medical conditions aged 18–50 years old (*n* = 450)
Charron-Prochownik et al. [36]	United States	Long-Term Effects of the Booster-Enhanced READY-Girls Preconception Counseling Program on Intentions and Behaviors for Family Planning in Teens with Diabetes	To examine 12-month effects of a booster-enhanced preconception counselling program (READY-Girls) on family planning for teen girls with T1DM and T2DM	Randomised controlled trial	Multicentre – 2 University hospitals diabetic clinics	Adolescent girls between 13 and < 20 years of age with either T1DM or T2DM for > 1 year (*n* = 109)
Nojomi et al. [60]	Iran	Contraceptive use by Iranian women with hypertension, diabetes or obesity	To determine the pattern of contraception use by women with DM, hypertension or obesity	Cross-sectional survey	Single centre – outpatient clinic of a university hospital	Women aged 18 to 53 years old with T2DM, hypertension or overweight/obesity (*N* = 264)
Perritt et al. [45]	United States	Contraception counseling, pregnancy intention and contraception use in women with medical problems: an analysis of data from the Maryland Pregnancy Risk Assessment Monitoring System (PRAMS)	To evaluate whether women with selected medical comorbidities are less likely than healthier women to report receiving contraceptive counselling during pregnancy and to report using contraception postpartum	Retrospective analysis from monitoring system survey	State-wide survey—Maryland Pregnancy Risk Assessment Monitoring System (PRAMS)	Postpartum women (*n* = 6,361)
DeNoble et al. [38]	United States	Receipt of Prescription Contraception by Commercially Insured Women with Chronic Medical Conditions	To assess differences in receipt of prescription contraception among women with and without chronic medical conditions	Retrospective study	Insurance claim records	Women aged 21 to 45 years old with medical condition, enrolled in commercial insurance company (*n* = 11,649)
Champaloux et al. (2015) [34]	United States	Contraceptive use among women with medical conditions in a nationwide privately insured population	To examine contraceptive use among women with selected medical conditions	Retrospective study	Nationwide healthcare claim database	Women aged 15 to 44 years old (*n* = 368,448) (10,903 with diabetes mellitus)
Mekonnen et al. [65]	Ethiopia	Contraceptive use in women with hypertension and diabetes: cross-sectional study in northwest Ethiopia	To assess contraceptive use and associated factors among diabetic and hypertensive women of reproductive age on chronic follow-up care at University of Gondar and Felege Hiwot Hospitals	Cross-sectional study	2 university hospitals	Women with diabetes and hypertension aged 15–49 years old (*n* = 392)
Osman et al. [64]	South Africa	Reproductive knowledge and use of contraception among women with diabetes	To assess the reproductive knowledge and use of contraception in women of reproductive age attending diabetes outpatient clinics	Prospective study	3 diabetic outpatient clinics	Women with diabetes mellitus aged 18 to 45 years old (*n* = 115)
Klingensmith et al. (2016) [43]	United States	Pregnancy Outcomes in Youth With Type 2 Diabetes: The TODAY Study Experience	To evaluate pregnancy outcomes during the Treatment Options for type 2 Diabetes in Adolescents and Youth (TODAY) study	Retrospective study	15 paediatric diabetes care clinics and practices	Youths aged 10–17 years old with Type 2 DM who participated in a RCT who reported pregnancies (*n* = 46)
Phillips Bell et al. [46]	United States	Chronic Diseases and Use of Contraception Among Women at Risk of Unintended Pregnancy	To assess whether three common chronic diseases (DM, cardiovascular disease, or asthma) were associated with use of contraception, according to various levels of effectiveness	Surveillance system data	State-wide surveillance—Florida Behavioral Risk Factor Surveillance System	Women aged 18–44 years old (*n* = 4,473)
Sereika et al. (2016) [50]	United States	Operationalizing and Examining Family Planning Vigilance in Adult Women With Type 1 Diabetes	To operationalize and describe Family Planning Vigilance (FPV) and examine the associations among FPV behaviours and diabetes self-care management (DSM) and health outcomes of women with T1DM	Retrospective cohort study	A university hospital	Women with T1DM who previously participated in adolescents’ preconception program (*n* = 102)
Schwarz et al. [47]	United States	Postpartum Care and Contraception provided to Women with Gestational and Preconception Diabetes in California’s Medicaid Program	To compare rates of postpartum care and contraception provided to women with gestational or preconception DM to women with no known DM	Retrospective cohort study	Medicaid claims state-wide	Women aged 15 to 44 years old under Medicaid program (*n* = 199,860)
Hibbert et al. [62]	Australia	Preconception care and contraceptive use among Australian women with diabetes mellitus	To explore preconception care practices and contraception use among women with DM	Cross-sectional survey	Tertiary referral centre	16–49 years old women with T1DM or T2DM (*n* = 215)
Britton et al. [25]	United States	Contraceptive use among women with prediabetes and diabetes in a US national sample	To estimate the relationship between contraceptive use and key measures of glucose dysregulation among women of reproductive age in a US national sample	Cross-sectional study	National Longitudinal Study of Adolescent to Adult Health	Sexually active women aged 24 to 32 years old (*n* = 5,548)
Law et al. [52]	Malaysia	Influences on the decision to use contraception among Sarawakian women with diabetes: a qualitative exploration	To explore the reasons for contraceptive use among Sarawakian women with DM	Qualitative study – in-depth interviews	A public primary care clinic	Sexually active, adult, pre-menopausal females (aged ≥ 18) with T2DM (*n* = 12)
Morris et al. [44]	United States	Description and comparison of postpartum use of effective contraception among women with and without diabetes	To describe contraceptive use and compare the effectiveness of contraceptive methods among postpartum women with and without DM	Cross-sectional study	Population-based data from the Pregnancy Risk Assessment Monitoring System (PRAMS)	Women who had recent live birth within 2–4 months (*n* = 93,754)
Britton et al. [33]	United States	Perceptions and Behaviors Related to Contraceptive Use in the Postpartum Period Among Women with Pregestational Diabetes Mellitus	To describe perceptions and behaviours related to contraception and preconception care and to test the association between these perceptions and contraceptive use in the postpartum period among women with pregestational DM	Cross-sectional study	Multicentre – 3 high-risk obstetric clinics	Postpartum women with diabetes mellitus (*n* = 55)
Disney et al. [39]	United States	Preconception counseling, contraceptive counselling, and long acting reversible contraception use in women with Type 1 diabetes: a retrospective cohort study	To describe the frequency of preconception counselling, contraceptive counselling, and LARC use by provider type and disease severity in reproductive age women with T1DM	Retrospective cohort study	Tertiary referral centre – data from Utah Enterprise Data Warehouse (EDW)	Women (16–49 years old) with T1DM (*n* = 541)
Hunter-greaves et al. [66]	Jamaica	Contraceptive practices in women with chronic medical conditions	To determine the contraceptive use in women with medical conditions at the University Hospital of the West Indies (UHWI)	Cross-sectional study	Single centre—medical outpatient departments in a university hospital	Females with various chronic medical conditions aged 18 to 44 years old (*n* = 260)
Leow et al. [53]	Malaysia	Patient perception of pre-pregnancy are and family planning among reproductive age female diabetes mellitus patients in a primary care clinic in Penang, Malaysia	To assess female diabetic patients’ perception of PPC and family planning prior to Pre-pregnancy care (PPC) care	Cross-sectional study	A public primary care clinic	Women with diabetes mellitus aged 18–45 years old (*n* = 67)
Horwitz et al. [42]	United States	Use of contraception before and after a diabetes diagnosis: An observational matched cohort study	To determine how a DM diagnosis affects contraception use	Retrospective cohort study	Using private insurance data from a large national private health insurance provider)	Non-pregnant women aged 15–49 years (*n* = 75,355 with diabetes, *n* = 7.5 million without diabetes)
Scott et al. [56]	United Kingdom	Pregnancy and contraception in women with Pre-Gestational diabetes in secondary Care– A questionnaire study	To establish the knowledge of women with pre-gestational DM about of the risks of diabetes in pregnancy, and investigate their pregnancy plans, contraceptive choices, and preparedness for pregnancy	Cross-sectional study	Multicentre—Diabetes clinics across a single NHS Trust	Women of reproductive age with diabetes mellitus (*n* = 96)
Feutry et al. [41]	United States	Contraception use and knowledge related to pregnancy in diabetic women	To assess diabetic patients’ knowledge about pregnancy and to describe their contraceptive use	Observational study	Single centre – university hospital	Women aged 18 to 40 years old with diabetes mellitus (*n* = 89)

Study populations varied where most of the studies (*n* = 27) did not specify the types of diabetes mellitus while six studies were specifically among women with Type 1 diabetes mellitus in specialist centre [10, 35, 39, 40, 49, 50] and three studies among Type 2 diabetes mellitus [43, 52, 60]. Nine studies reported diabetes mellitus with other selected medical conditions [37, 38, 45, 46, 51, 54, 60, 65, 66] and another six studies include all women within reproductive age group in the analysis [25, 34, 37, 42, 47, 48]. Two studies targeted specifically postpartum women with diabetes mellitus [33, 44]. Four studies were specifically among adolescents with diabetes mellitus [35, 36, 40, 49]. The age ranges to define ‘reproductive age’ in the studies varied, with a study used a limited range of 24 to 32 years old [25] and other studies with wider ranges i.e., 15 to 44 years old, 18 to 44 years old and 15 to 45 years old.

One study made comparison of the proportion of family planning usage based on disease status; diagnosed diabetes, undiagnosed diabetes, uncontrolled diabetes and no diabetes [25]. Six studies have explored pregnancy intention [33, 41, 45, 51, 53, 56] but only one study descriptively investigated the relationship between pregnancy intention in relation to contraceptive use of the study participants [56]. Most of the studies described the usage of family planning practice by types of methods (*n* = 26) [10, 25, 33–35, 38, 40–42, 44, 47–51, 54, 56–62, 64–66] and eight studies categorized the methods according to level of effectiveness [25, 41, 46, 48, 50, 53, 56, 62]. Only one study studied specifically the use of long-acting reversible contraception (LARC) among women with diabetes mellitus [39]. Five studies described the barriers of family planning usage or reasons of contraceptive non-use [33, 41, 52, 58, 65]; two of them through qualitative exploration [52, 58].

### Results of individual sources of evidence

Main findings of the studies including the proportion of women using contraceptive methods and factors influencing family planning behaviours are summarised in Table 5.

**Table 5:** Summary of factors influencing family planning behaviours

Author (Year)	Proportion of women with DM using contraceptive methods	Information on methods used	Factors influencing family planning behaviours				
			Individual level	Interpersonal level	Community level	Institutional level	Policy level
Falsetti et al. [54]	62%	• Types of methods55 • Dual methods usage	–	–	–	–	–
Chuang et al. [37]	74.20%	–	• Age (−) • Ethnicity • Marital status (−) • Education attainment ( +) • Health insurance status ( +)	–	–	–	–
Diabetes and Pregnancy Group, [63]	61%	Types of methods	• Knowledge of risk of congenital malformations ( +)	–	–	–	–
Napoli et al. [61]	89.4%	Types of methods	Characteristics of oral contraceptive user: • Younger • Slimmer • Earlier age at first intercourse • Highest living in north • Lowest living in south • Higher education	Geographic region	Service provider: • Diabetologist • Gynaecologist	–	–
Charron-Prochownik et al. [35]	22.60%	Types of methods	–				
Shawe et al. [57]	OR 0.83	Types of methods	Likelihood of methods based on diagnosis of diabetes and type of diabetes	–	–	–	–
Mazaheri et al. [59]	73%	Types of methods	–	–	–	–	–
Vahratian et al. [51]	61.2%	Types of methods	• BMI (−) • Age (−) • Ethnicity—non-Hispanic black ( +) • Cohabitating (−) • History of infertility treatment (−) Desired or ambivalent about pregnancy (−)	–	–	–	–
Schwarz et al. [49]	14% among all respondents 57% among ever sexually active	Types of methods	–	–	–	–	–
Schwarz et al. [48]	37.7%	• Types of methods • Effectiveness of methods • Highly effective • Moderately effective • Less effective	Age—older age—higher % intrauterine (IUD) usage	–	–	–	–
Shawe et al. [58]	66% (questionnaire respondents) 11 out of 16 interview participants	Types of methods	Healthcare providers factors: • Diabetes specialist felt they were unqualified to give contraception advices • Conversation about contraception not considered as part of regular consultation Choice of methods—diabetic women prefer 'natural' methods	–	–	–	–
Manaf et al. [54]	28.8%	Types of methods	• Age (−) • Ethnicity (x) • Employment status (x) • Education attainment ( +) • Parity ( +) • Perception on contraception ( +) • History of perinatal death (x)	–	–	Types of health facility: • Health clinics • Hospital specialist clinics	–
Charron-Prochownik et al. (2013) [36]	36% in control group and 64% in intervention group had at least 1 episode of unprotected sex	–	Intervention vs control	–	–	–	–
Nojomi et al. [60]	58.8%	Types of methods	• Types of most common methods differ after diagnosis of diabetes • Higher percentage of using withdrawal or no method: • Higher literacy parity (more in parity < 2 and > 4)	–	–	–	–
Perritt et al. [45]	24.6% (preconception) 77% (postpartum)	–	• Presence of medical condition • Receipt of contraceptive counselling ( +)	–	–	–	–
DeNoble et al. [38]	34.2%	Types of methods	• Age (−) • Socioeconomic status (x) • Total outpatient visits (−) • Cervical cancer screening ( +) • Types of medical conditions (x)	–	–	–	–
Champaloux et al. [34]	44%	Duration of action • Short-acting • Long-acting Irreversibile (sterilisation)	• Age (−) Presence of medical conditions	–	–	–	–
Mekonnen et al. [65]	53.8	Types of methods	• Age • Income • Disease control • Having living children ( +) Contraceptive counselling receipt	–	–	–	–
Osman et al. (2015) [64]	67%	Types of methods	• Parity (x) • Education (x) • Emotional support (x) • Marital status ( +)	–	–	–	–
Holmes et al. [55]	44.4% in pre-DVD group	–	–	• Pre and post DVD intervention (x)	–	–	–
Klingensmith et al. [43]	4.8%	–	–	–	–	–	–
Phillips Bell et al. [46]	89.8%	Effectiveness of methods • Effective/highly effective • Less effective No method	Types of medical condition—and the likelihood of using more effective methods	–	–	–	–
Sereika et al. [50]	50%	• Types of methods	Women who are vigilant: • More likely to have preconception care earlier • More likely to use more effective family planning • More likely to report better health outcomes	–	–	–	–
Schwarz et al. [47]	47.8%	• Types of methods • Effectiveness of methods - weighted summary measure	• Presence of diabetes—likelihood of permanent contraception	–	–	–	–
Hibbert et al. [62]	75.3%	• Types of methods • Effectiveness of methods	–	–	–	–	–
Britton et al. [25]	71.2%	• Types of methods • Effectiveness of methods • More effective • Less effective • None	• Non-Hispanic Black Women ( +) • Education attainment ( +) • BMI (−) • Health insurance status ( +)	–	–	Access to care (+)	–
Law et al. [52]	–	–	• Perceived likelihood of becoming pregnant • Desired family size • Perceived health risks associated with diabetes Social implications of becoming pregnant (job disruption and economic impact)	• Opinions of significant others	–	–	–
Morris et al. [44]	82%	• Types of methods • Reversibility of methods	• For sterilisation age (+) Education (−) Previous live births ( +) Recent unintended pregnancy ( +) Government or no health insurance ( +) Caesarean delivery ( +) • For LARC Diagnosed with diabetes ( +) Younger age < 19 yo ( +) Education ( +) Ethnicities (+ hispanic or other ethnicities) vs non-Hispanic white Previous live births ( +) Recent unintended pregnancy ( +) Government or no health insurance ( +) Caesarean delivery ( +)	–	–	–	–
Britton et al. [33]	56%	• Types of methods: • Procedure/ prescription methods • Non-prescription methods	• Perception Perceived benefit ( +) Perceived barriers (x) Self-efficacy (x) • Patient characteristics Age Ethnicity Religion Education Primigravida Health insurance type Type of diabetes Age at diagnosis (x) • Pregnancy planning status (x)	–	–	–	–
Disney et al. [39]	13% (LARC)	• Specifically on long-acting reversible contraception (LARC) usage	• Age (−) • Presence of advanced disease complication (x) • Frequency of visits (+) • Receipt of preconception and contraceptive counselling (+)	–	–	Types of service providers • Fetomaternal specialists • Obstetricians & Gynaecologists • Endocrinologists • Primary care provider	–
Hunter-greaves et al. [66]	60.4%	• Types of methods	• Marital status (+) • Previous adverse pregnancy outcome: Neonatal death (−) Pregnancy-induced hypertension (+) Previous ICU admission ( +)	–	–	–	–
Leow et al. [53]	59.8%	Effectiveness of methods based on Pearl Index	• Sociodemographic characteristics • Marital status • Pregnancy intention • Knowledge on contraceptive effectiveness	–	–	–	–
Horwitz et al. [42]	11.9%	Types of methods	• Age—younger group more pronounced negative relationship between diagnosis and usage • Ethnicity (x) Diagnosis of diabetes (-)	–	–	–	–
Scott et al. [56]	75%	• Types of methods • Efficacy of methods • Low efficacy • Moderate efficacy • High efficacy	• Pregnancy intention and efficacy of methods (28% who were not planning used low efficacy methods)	–	–	–	–
Feutry et al. [41]	72.1%	• Types of methods • Effectiveness of methods based on Pearl Index • Usage of contraindicated methods	• Pregnancy intention (x) • Type of diabetes (lower in T2DM)	–	–	–	–

The following annotation represented when the factors were tested statistically, otherwise the factors were only reported descriptively:

( +) positively associated

(−) negatively associated

(x) not associated

### Measures of family planning usage among women with diabetes mellitus

Majority of the studies defined family planning practice as binary outcome whether any contraceptive methods were used or not used. In relation to temporal measure, most of the studies described family planning usage as ‘currently using’, but three studies specify the duration of usage within the last one to three years [38, 62, 64]. One study categorized family planning behaviours based on a composite score which take into consideration contraception use, receipt of preconception counselling and initiation of discussion with healthcare professionals [50].

Eight studies explored and classified the usage of contraception based on its effectiveness or efficacy as summarized in Table 4. Phillips-Bell et al. [46] found that women without diabetes were more likely to use more effective methods compared to women with diabetes which was parallel with findings from Britton et al. [25] and Schwarz et al. [48] which reported that women with diabetes mellitus was less likely to practice highly effective methods. Meanwhile, Morris & Tepper [44] reported that women with diabetes were more likely to use effective and long-acting methods. Leow et al. [53] found that only small proportion of women with diabetes were using highly effective methods and it did not correspond with their risk perception.

### Prevalence and likelihood of family planning usage among women with diabetes mellitus

Among the studies that compared the likelihood of family planning usage among women with diabetes mellitus compared to women without diabetes, six studies reported that women with diabetes mellitus were less likely to use family planning methods [25, 38, 45, 48, 54, 57]. Only one study found that the likelihood of practicing family planning methods among women with diabetes mellitus were not significantly different compared to women without diabetes [37].

The prevalence of family planning methods used by women with diabetes mellitus varied across the reviewed literature ranging from 4.8 to 89.8% among the studied population.

### Reasons for contraceptive non-use among women with diabetes mellitus

In studies that provided additional information on the reasons of contraceptive non-use, the most commonly described reasons were the misconceptions on the safety of contraceptive usage with the presence of diabetes mellitus, fear of side effects, and perceptions of reduced fertility with diabetes mellitus. Lack of preconception or contraceptive counselling provision during visits for diabetes care were also commonly reported reasons [37, 41, 65]. These reasons were elicited from quantitative studies using self-administered questionnaires. One of the studies utilized free-text field for this measure [33] while two other studies did not specify how reasons for non-use were addressed in the survey [41, 67].

### Factors influencing family planning usage

The explanatory variables or determinants of family planning usage were grouped according to different levels (i.e., individual levels, interpersonal levels, community levels, institutional levels and policy levels) that were commonly described in family planning usage among general population. The findings are summarized in Table 4. Only five studies included beyond individual-level factors. One study addressed differences in family planning practice among women with diabetes mellitus in different regions [61] and three studies included types of healthcare facilities and service providers which were considered as institutional-level factors [39, 54, 61]. A qualitative study reported interpersonal-level factor as described below.

#### Individual-level factors

Sociodemographic characteristics that were frequently described to influence family planning behaviour in the studies among women with diabetes mellitus include age, ethnicity, marital status and education attainment. Age was shown to have negative association with family planning practice, where the younger age group were more likely to use contraception while only Morris et al. [44] reported otherwise. Horwitz [42] and Manaf [54] found no significant association between ethnicity and family planning non-users while three other reports found significant association between family planning usage and specific ethnic groups [25, 44, 51]. Education attainment of the women was another commonly reported factors that were positively associated with family planning usage and the findings were quite consistent [25, 33, 37, 44, 54, 60, 61]. However, when specifically studied for sterilisation, women with lower education attainment were more likely to have the procedure performed [44]. Women with middle- and high-level household income were more likely to practice family planning [65]. Along the same line, qualitative exploration also found that family planning usage were influenced by social implications of pregnancy including financial burden and career disruption [52].

Significant reproductive history that predicts family planning usage among women with diabetes mellitus includes parity and history of previous adverse pregnancy outcome. Mixed findings were reported in relation to parity and its association with family planning usage [25, 54, 60, 64, 65]. Qualitative exploration among women with diabetes mellitus reported that their experience in previous pregnancy influenced their decision to practice family planning methods where they were more inclined to practice when they had ‘previous difficult labour’ [52].

Women with diabetes mellitus who previously received contraceptive counselling were significantly more likely to practice family planning [39, 45, 65]. Knowledge on risk of congenital malformations were reported to be positively associated with family planning usage [63]. A descriptive study explored knowledge and usage of family planning methods based on its effectiveness. Women with diabetes mellitus in that study were found to have inadequate knowledge where their pregnancy intention and risk perception did not correspond to the usage of effective contraception methods [53]. Positive perception towards contraception were found to be positively associated with its usage [25, 54].

Association of disease control with family planning usage were explored in two studies. Mekonnen, Woldeyohannes & Yigzaw [67] reported that women with controlled condition were four-times more likely to practice contraception, while Britton et al. reported that nearly half of women with suboptimal HbA1c practiced less effective contraceptive methods [25]. Four other studies also grouped women with diabetes mellitus according to their disease control but their association with family planning behaviours was not explored.

#### Interpersonal-level factors

Only one of the study explored this interpersonal aspects where in-depth interviews among women with diabetes mellitus reported that opinions of significant others including friends, respected older female relatives, health care personnel, or religious leaders influenced their decision in family planning [52]. Desired family size was also influenced by partners and directly influenced the usage of family planning methods.

#### Institutional-level factors

A study compared the types of facility where the women received care by either in health clinics or in hospitals [54]. This study found that contraceptive non-use was significantly more common among women who received care in the health clinics as compared to those who received treatment in the hospital. Another study compared the types of healthcare providers who provided the care to the women with diabetes mellitus [39]. Not surprisingly women who were seen by gynaecologist or fetomaternal specialists were more likely to be counselled on the use of family planning. A study in Italy described the proportion of oral contraceptive pills prescribed by different specialists where majority were prescribed by gynaecologists [61]. One study categorized access to healthcare and studied its relationship with family planning usage found that it was associated with the usage of effective contraception [25].

#### Community-level factors

Only one study addressed community-level factor by comparing oral contraceptive usage among women with diabetes mellitus from different regions in Italy where they reported significant regional difference in usage prevalence [61].

## Discussion

### Summary of evidence

This scoping review identified 35 studies addressing family planning practice among women with diabetes mellitus published between 2003 and January 2022 which revealed family planning behaviours that vary throughout the studies. This review also revealed inconclusive estimates on the proportion of women with diabetes mellitus who were using family planning methods with a huge range of prevalence reported. This may be attributed to the widely-diverse study populations and settings. Study populations included in this review ranged from all women of reproductive age based on a nationwide survey to a specific group of adolescents with a specific type of diabetes mellitus in a tertiary setting. The types of diabetes mellitus may have a different impact on family planning behaviours [41]. Variation in the age group of study participants also portrays the complexity in determining the group of women at risk of pregnancy even though WHO has defined reproductive age group as 15 to 49 years old [68]. Approach to prevent unintended pregnancies also should be tailored to different age group of girls or women.

As almost half of the studies were carried out among women with diabetes mellitus in specialist centres, knowledge on family planning among women in general practice or primary care was still limited. Majority of women with uncomplicated diabetes mellitus received care in the primary care setting. Manaf [54] reported that women who received their treatment in health clinics were less likely to use contraception than women who received their treatment in hospital specialist clinics. This is possibly due to different disease profile and level of diabetic complications amongst the women who received specialist care in hospitals.

The majority of the studies had focused on descriptive characteristics of family planning behaviours where there is knowledge gap remains particularly with regards to associations of family planning usage and its influencing factors. Exploration of factors influencing family planning behaviours among women with diabetes mellitus were also very limited to sociodemographic and individual-level characteristics. There were very limited studies that acknowledged the determinants beyond individual levels which represents a gap in the knowledge. This is because studies among general population have established multilevel influences in determining family planning usage [69]. The single qualitative study included in this review can be considered as an important initial exploration as it revealed that interpersonal factors play a big role in the decision to practice family planning, but the findings should be interpreted with caution as the study is done in a single state in Malaysia with different ethnic proportion from the rest of the country [52]. This highlighted the areas with insufficient knowledge that limits our understanding on the factors that influence family planning behaviours among this specific population.

Designs of the studies included in the review may also give rise to inaccuracy of family planning behaviour measurement. As most of the studies were cross-sectional, the information on family planning behaviours were only measured based on their current usage of the contraceptive methods. However, three studies explored the usage or prescription of family planning over a duration of time. Nine of the studies were done retrospectively by reviewing medical databases and insurance claim records which may underestimated family planning usage when documentation omission or errors frequently occurred [39]. Utilisation of this secondary data enabled analysis of larger number of women. However, the family planning usage may be underestimated as it only captured prescription-based methods and methods covered by the insurance plan [34, 38]. Prescription of contraception by the healthcare providers also did not provide information whether the women actually used the contraception and reliance on insurance claims record had limited information on non-prescription methods [38].

Preventing unintended pregnancy among women with poorly controlled diabetes mellitus or with complications is the main strategy to prevent morbidity and mortality, but only four studies included information on disease control in their study [25, 39, 50, 61]. This may represent inadequate attention given to the main objective of pregnancy planning among high-risk women. Other relevant clinically-relevant evidence in women with diabetes mellitus especially when conditions were not optimized is the effectiveness of family planning methods used, but limited number of studies explored family planning usage among this population according to their effectiveness. As the compliance with usage is another important factor in preventing unintended pregnancy, the duration of usage and continuation rate of the methods were valuable but were not measured in any of the studies. Only one study reported compliance with methods among the participants where half of the women who were on oral contraceptives claimed that they regularly missed the pills [41]. Most studies were based on self-reported data on the status of disease during survey. Therefore, reporting bias may have been introduced [25, 37, 45, 46, 51].

Only four studies reported family planning usage as the outcome after an interventional study [36, 43, 50, 55]. This indicates that there is inadequate evidence on effective strategies to improve family planning uptake among women with diabetes mellitus.

Future studies with more clinical context in relation to family planning behaviours and diabetes mellitus including types of diabetes, disease control, presence of diabetes complications would be beneficial to target high-risk women in prevention of unintended pregnancies. Knowledge gaps at system-level limit the availability of evidence-based strategies that can be utilized by health practitioner or policymakers. System-level evidence is also needed to address further targeted innovations in family planning service provision to women with diabetes mellitus.

### Strengths and limitations

This is the first published scoping review to explore the evidence surrounding family planning behaviours among specific population of women with diabetes mellitus. This review offers a comprehensive overview of the available evidence on family planning behaviours of women with diabetes mellitus which may contribute to the improvement of family planning or preconception service provision to improve the outcome of mothers and their children.

The limitations of this scoping review are also acknowledged. Stakeholder consultation has been suggested as one of the distinctive components of a scoping review that will give additional insight from the stakeholders’ perspectives. However, this was not carried out in our scoping review. Future studies that incorporate this valuable element is recommended to add methodological rigor and enhance the applicability of the review [16]. Apart from that, studies published in other languages were not included which may have contributed to more culture-specific predictors of family planning behaviours. However, the studies included in the review were from various regions and these studies could have attempted to represent studies in the region. This review also does not incorporate the critical appraisal of the evidences (Additional file 1).

## Conclusion

The evidence on family planning behaviours among women with diabetes mellitus was limited to the sociodemographic factors. Future studies with more clinical and contextual factors are needed to guide the strengthening of family planning services for high-risk women specifically those with diabetes mellitus and limited knowledge on clinical and contextual factors that influence the behaviour.

### Supplementary Information

**Additional file 1.** Search strategy example.

## Data Availability

Original articles reviewed in this manuscript are available on PubMed, EBSCO and OVID databases.

## Abbreviations

DM: Diabetes mellitus
PRISMA-ScR: Preferred reporting items for systematic reviews and meta-analyses extension for scoping reviews
ICU: Intensive care unit
IUD: Intrauterine device
LARC: Long-acting reversible contraception
LMIC: Low-middle income countries
PCC: Population-concept-context
PRAMS: Pregnancy risk assessment monitoring system
STDs: Sexually transmitted diseases
T2DM: Type 2 diabetes mellitus
WHO: World Health Organization
