# Supplementary material for: Family planning behaviours among women with diabetes mellitus: a scoping review
Source: Eur J Med Res. 2024 Jan 11;29:41. doi: 10.1186/s40001-023-01626-1 (PMC10782608; doi:10.1186/s40001-023-01626-1)
Supplement: Supplementary file 1 — Additional file 1. Search strategy example. [file 40001_2023_1626_MOESM1_ESM.docx]

**Supplementary File 1: Search Strategy**

**Search strategy for PubMed (Search conducted on 16 March 2022)**

| **Number** | **Search terms** |
| --- | --- |
| #1 | "family planning"[Title/Abstract] OR "contraceptive"[Title/Abstract] OR "contraception"[Title/Abstract] |
| #2 | diabetes[Title/Abstract] OR diabetic[Title/Abstract] |
| #3 | ("family planning"[Title/Abstract] OR "contraceptive"[Title/Abstract] OR "contraception"[Title/Abstract]) AND ("diabetes"[Title/Abstract] OR "diabetic"[Title/Abstract]) |
| #4 | (("family planning"[Title/Abstract] OR "contraceptive"[Title/Abstract] OR "contraception"[Title/Abstract]) AND ("diabetes"[Title/Abstract] OR "diabetic"[Title/Abstract])) AND (2001:2022[pdat]) |

**Note: Search in PubMed automatically searched for MeSH terms.**
